# Eltrombopag as an Allosteric Inhibitor of the METTL3-14 Complex Affecting the m^6^A Methylation of RNA in Acute Myeloid Leukemia Cells

**DOI:** 10.3390/ph15040440

**Published:** 2022-04-01

**Authors:** Je-Heon Lee, Namjeong Choi, Subin Kim, Mi Sun Jin, Haihong Shen, Yong-Chul Kim

**Affiliations:** School of Life Sciences, Gwangju Institute of Science and Technology (GIST), Gwangju 61005, Korea; jhl@gm.gist.ac.kr (J.-H.L.); njchoi@gist.ac.kr (N.C.); ksb1201@gm.gist.ac.kr (S.K.)

**Keywords:** eltrombopag, METTL3-14, allosteric inhibitor, acute myeloid leukemia

## Abstract

N^6^A-methyladenosine (m^6^A) post-transcriptional modification, the most abundant internal RNA modification, is catalyzed by the METTL3-14 methyltransferase complex. Recently, attention has been drawn to the METTL3-14 complex regarding its significant roles in the pathogenesis of acute myeloid leukemia (AML), attracting the potential of novel therapeutic targets for the disease. Herein, we report the identification and characterization of eltrombopag as a selective allosteric inhibitor of the METTL3-14 complex. Eltrombopag exhibited selective inhibitory activity in the most active catalytic form of the METTL3-14 complex by direct binding, and the mechanism of inhibition was confirmed as a noncompetitive inhibition by interacting at a putative allosteric binding site in METTL3, which was predicted by cavity search and molecular docking studies. At a cellular level, eltrombopag displayed anti-proliferative effects in the relevant AML cell line, MOLM-13, in correlation with a reduction in m^6^A levels. Molecular mechanism studies of eltrombopag using m^6^A-seq analysis provided further evidence of its cellular function by determining the hypomethylation of leukemogenic genes in eltrombopag-treated MOLM-13 cells and the overlapping of the pattern with those of METTL3-knockdown MOLM-13 cells. In conclusion, eltrombopag was first disclosed as a functional METTL3-14 allosteric inhibitor in AML cells, which could be utilized for the further development of novel anti-AML therapy.

## 1. Introduction

Of the over 170 RNA modifications occurring in diverse cell systems, N^6^-methyladenosine (m^6^A) is the most prevalent and abundant internal modification of particular mRNAs in the long exon and 3′ untranslated region (3′ UTR) near the stop codon of mRNA with the consensus sequence RRACH (R = A or G, H = A, C or U) [1,2,3,4]. The mechanisms and functions of this modification have been recently explored, revealing regulatory functions in the fate of mRNA by affecting its nuclear export, splicing, stability, and translation efficiency [5,6,7]. Consequently, the m^6^A modification turned out to be closely involved in multiple biological processes, including self-renewal, development, metabolism, homeostasis, and immunity [8,9,10,11,12,13]. As the components play various roles in m^6^A modification, the N^6^-methyltransferase complex (writers) and demethylases (erasers) catalyze the reversible regulatory functions by methylation and demethylation, respectively, and the resulting products are recognized by m^6^A binding protein (readers) to further forward biological processes [14,15,16]. 

Among them, the N^6^-methyltransferase complex consists of catalytic components, methyltransferase-like protein 3 (METTL3) and 14 (METTL14), and other regulatory m^6^A-associated complexes, such as Wilms tumor 1-associated protein (WTAP), Vir-like m6A methyltransferase associated (VIRMA), Cbl protooncogene like 1 (CBLL1), RNA-binding motif 15 (RBM15), and zinc finger CCCH-type containing 13 (ZC3H13) [17,18,19,20]. The individual METTL3 and METTL14 exhibit relatively weak catalytic activity in vitro, but their heterodimeric complex has a much higher activity, which is the physiologically relevant form in the nucleus [17,21]. The crystal structure of the heterodimeric complex of METTL3-14 demonstrated that METTL3 is mainly responsible for catalytic function, transferring a methyl group from S-(5′-adenosyl)-_L_-methionine (SAM) to the N^6^-amine of adenine, while METTL14 promotes METTL3 catalytic activity by providing an RNA-binding surface composed of positively charged residues [22,23,24].

Recent studies of METTL3 and METTL14 in cancers have shown that they are closely associated with the processes involved in the proliferation, apoptosis, metastasis, and differentiation in the progression of various human cancers [25,26,27]. In particular, acute myeloid leukemia (AML), one of the most common types of leukemia with diverse genetic and molecular abnormalities in adults, expresses higher levels of METTL3 and METTL14 compared with other cancer types in the analysis of The Cancer Genome Atlas (TCGA) dataset [28,29,30,31]. The significance of METTL3 and METTL14 in AML progression was further investigated by knockdown experiments of the AML cell line, which resulted in the induction of apoptosis and cell differentiation [30,32,33]. The role of METTL3 in tumorigenesis has been reported to promote the translation of c-MYC, BCL2, and PTEN in MOLM-13, a human AML cell line [32]. Another report showed that METTL3 is recruited at the transcription initiation site (TSS) by the CAATT enhancer binding protein zeta (CEBPZ), which resulted in the enhanced translation of oncogenes SP1 and SP2 to maintain the leukemic state [33]. In addition, METTL14 has been reported to play a critical oncogenic role by increasing the mRNA stability and translational efficiency of MYB and MYC through the m^6^A modification in the NB4 human AML cell line [30]. Therefore, METTL3 and METTL14 have drawn increased attention as attractive new therapeutic targets for the treatment of AML. 

**Figure 1:**
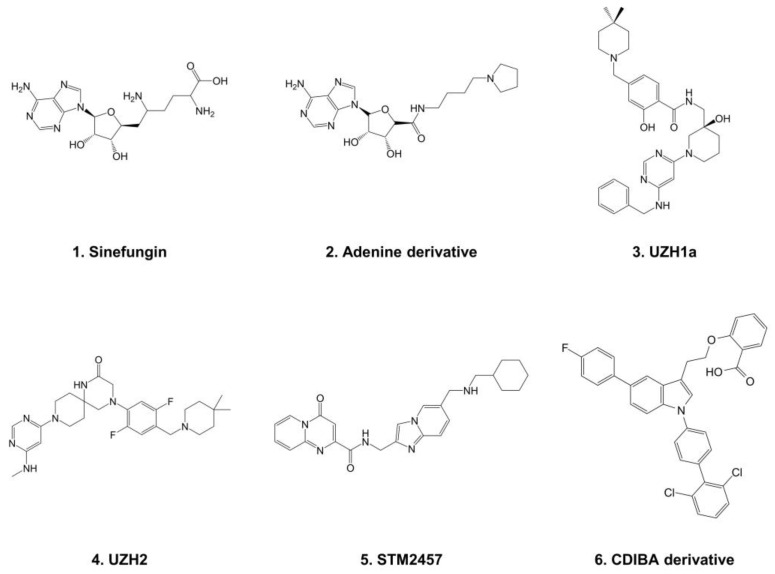
Chemical structures of current METTL3-14 inhibitors.

As such, chemical inhibitors of METTL3-14 have been developed for the discovery of novel mechanism-based anti-AML therapies. At first, the enzyme reaction product S-(5′-adenosyl)-L-homocysteine (SAH) and non-selective nucleoside analog sinefungin (**1**) were reported as the inhibitors of METTL3-14 [34,35]. Until recently, only a few SAM competitive inhibitors of METTL3 have been developed, including adenine derivatives (**2**) [36], UZH1a (**3**) [37], UZH2 (**4**) [38], and STM2457 (**5**) [39] (Figure 1). Although some SAM competitive inhibitors have shown selective inhibition profiles in the methyltransferase panel screening, allosteric inhibitors are preferred to avoid possible nonselective inhibitions, since the SAM binding region was conserved in most of the methyltransferase enzyme family [40,41,42]. In that sense, our group recently reported 4-[2-[5-chloro-1-(diphenylmethyl)-2-methyl-1H-indol-3-yl]-ethoxy]benzoic acid (CDIBA) derivatives (**6**) as the first allosteric inhibitor of METTL3-14 [43]. 

In this article, we report the discovery of eltrombopag as another allosteric inhibitor of METTL3-14 identified by screening the drug library from the Korea Chemical Bank. Eltrombopag was first reported as a thrombopoietin receptor (TPO-R) agonist for the treatment of immune thrombocytopenia (ITP), and approved by the U.S. Food and Drug Administration for the treatment of chronic ITP and aplastic anemia in 2008 and 2014, respectively [44,45,46]. Herein, we present the identification and characterization of eltrombopag as a METTL3-14 allosteric inhibitor, including the prediction of a putative binding site, anti-proliferative effects on AML cell lines, and an analysis of the influences on the m^6^A abundance on a cellular level. Furthermore, the molecular mechanism of eltrombopag was confirmed using m^6^A-seq analysis.

## 2. Results and Discussion

### 2.1. Enzyme Assay and Hit Identification of Eltrombopag as a METTL3-14 Inhibitor

To aid in the discovery of METTL3-14 inhibitors, we developed an enzyme-based bioluminescence assay protocol measuring the METTL3-14 enzymatic reaction product, SAH, by converting it to adenosine triphosphate (ATP), which could be quantitatively detected by the luminescence-mediated luciferase enzymatic activity. Then, the Michaelis–Menten of the kinetic parameters of METTL3-14 with SAM and RNA substrates were determined as the Km values with 98.6 nM and 70.5 nM for SAM and RNA, respectively (Figure S1), which are similar to those of the previously reported METTL3-14 assay systems [34,35]. 

**Figure 2:**
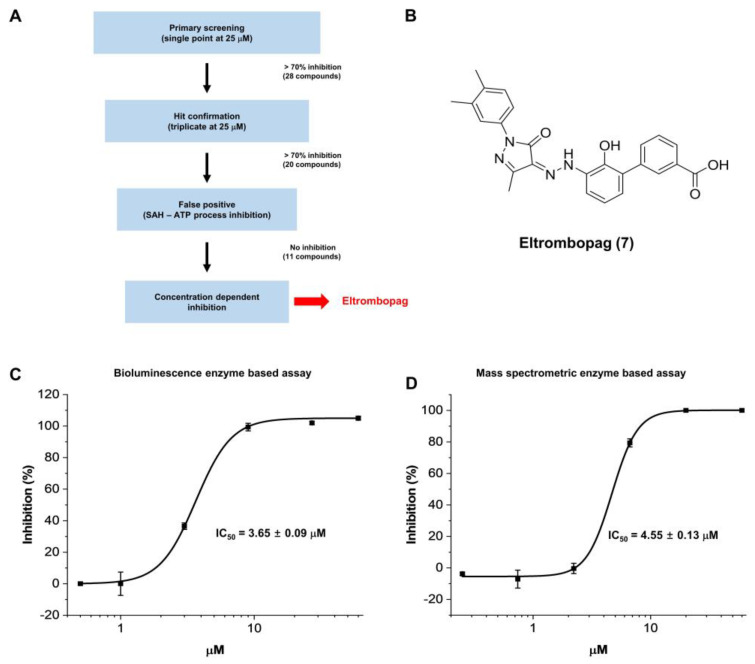
Identification of Eltrombopag as a novel inhibitor of the METTL3-14 complex through library screening. (**A**) Eltrombopag was discovered as a METTL3-14 inhibitor through the described procedure for screening of drug compound library. (**B**) Chemical structure of eltrombopag. (**C**) Concentration-dependent enzymatic inhibitory activity of eltrombopag on METTL3-14 was confirmed in a bioluminescence enzyme-based assay system with an IC_50_ value of 3.65 μM. (**D**) Enzymatic inhibitory activity of eltrombopag on METTL3-14 was confirmed in the mass spectrometric enzyme based assay system with an IC_50_ value of 4.55 μM.

Using the verified assay system, the drug compound library (2300 compounds) from the Korea Chemical Bank was initially screened at 25 μM, identifying 20 hit compounds with more than 70% inhibition (Figure 2A). After the exclusion of 9 false-positive compounds, which inhibit the coupled enzyme reaction processes converting SAH to ATP (Figure S2A), and further dose-response titration experiments of the true positive 11 compounds, eltrombopag (**7**) was discovered as the most potent final hit compound with an IC_50_ value of 3.65 μM (Figure 2B,C)—which was previously reported as a thrombopoietin receptor agonist [44]. The similar inhibitory potency (IC_50_ = 4.55 μM) of eltrombopag at METTL3-14 was additionally confirmed in a counter assay system using mass spectrometric analysis that measured the m^6^A level of the single strand RNA substrate (Figure 2D). Moreover, the METTL3-14 inhibitory activity of eltrombopag was further clarified in the centrifugation experiment, which could exclude the possible false-positive inhibition induced by colloidal aggregation (Figure S2B). 

**Figure 3:**
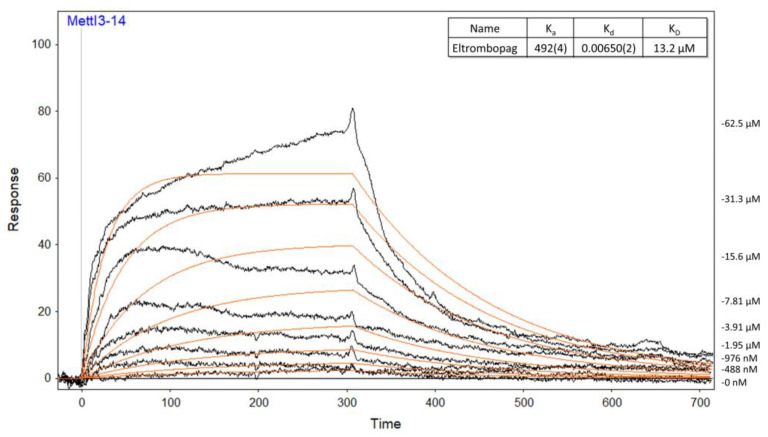
The Binding affinity of eltrombopag was evaluated by measuring the equilibrium dissociation constant (K_D_ value) to the METTL3-14 complex as 13.2 μM in surface plasmon resonance assay.

To determine whether eltrombopag directly binds to the METTL3-14 complex, the dissociation constant between eltrombopag and the METTL3-14 complex was measured using surface plasmon resonance (Figure 3). As a result, the binding of eltrombopag was observed in a concentration dependent manner with 13.2 μM of the calculated K_D_ value, as shown in Figure 3, demonstrating that the inhibition of eltrombopag on the METTL3-14 enzyme activity could occur by direct binding. To explore the selectivity profile of eltrombopag for METTL3-14, seven other methyltransferase family enzymes were investigated, resulting in a relatively low inhibitory activity of 10 μM eltrombopag on the SAM-dependent methyltransferases listed in Table 1. Taken together, our data suggest that eltrombopag has selective inhibitory activity at the METTL3-14 complex by direct binding to the enzyme protein. 

### 2.2. Mode of Enzyme Inhibition and Predicted Binding Mode of Eltrombopag in METTL3-14

**Figure 4:**
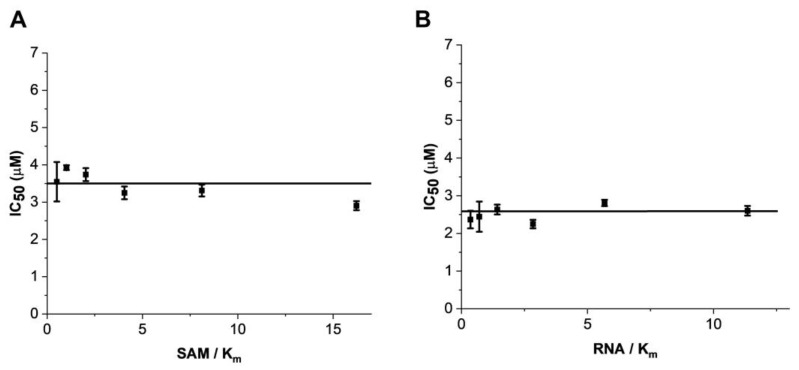
Studies on the mechanism of action showed that eltrombopag inhibited the METTL3-14 complex in a noncompetitive manner. (**A**) IC_50_ values of eltrombopag at increasing concentrations of SAM ranging from 50 to 1600 nM at fixed concentrations of RNA substrate (2 μM). (**B**) IC_50_ values of eltrombopag at increasing concentrations of RNA substrate ranging from 25 to 800 nM at fixed concentrations of SAM substrate (3 μM).

To understand the mechanism of action of eltrombopag in the METTL3-14 complex, the tendency of its inhibitory activity was determined from a variation of IC_50_ values of eltrombopag depending on the different substrate concentrations. As shown in Figure 4, the IC_50_ values of eltrombopag were not affected even when the concentrations of the SAM and RNA substrates varied from 50 to 1600 nM and from 25 to 800 nM, respectively. These results suggest that eltrombopag acts as a noncompetitive inhibitor, possibly by binding to the allosteric site of METTL3-14 rather than to the catalytic active sites of the SAM or RNA substrate binding pockets. Although allosteric modulators generally show lower binding affinity than orthosteric ligands, they could have other advantages, such as potentially higher selectivity and fewer side effects than orthosteric ligands [47]. Therefore, further optimization studies of eltrombopag, to improve the binding affinity and inhibitory activities based on its binding mode in METTL3-14, could achieve distinguished novel inhibitors with higher selectivity than other SAM competitive inhibitors.

**Figure 5:**
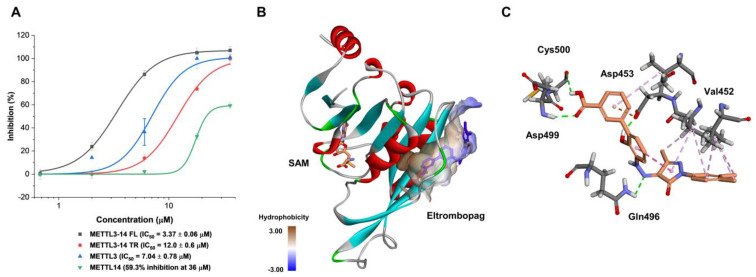
Predicted binding mode of eltrombopag with the METTL3 crystal structure. (**A**) Inhibitory activities of eltrombopag in various METTL3-14 enzyme forms (METTL3-14 FL: full length of METTL3-14, METTL3-14 TR: truncated form of METTL3-14 consisting of a methyltransferase domain). (**B**) Hydrophobicity surface view at the predicted allosteric binding pocket with the docked eltrombopag into METTL3 crystal structure (PDB: 5IL1 A chain). (**C**) Docking pose of eltrombopag with METTL3 (the green dashed lines represent hydrogen bond).

Next, in order to predict the allosteric binding site in which eltrombopag might interact and bind, its inhibitory activities were evaluated first at various enzyme forms of METTL3-14, such as each single form of METTL3 and METTL14 and the truncated form of METTL3-14 with only the methyltransferase domain (Figure 5A). As a result, eltrombopag maintained its inhibitory activity with an IC_50_ value of 7.04 μM in the single subunit enzyme form of METTL3, whereas a dramatically reduced partial inhibition was observed in the single subunit enzyme form of METTL14 with 59.3% at 36 μM. The profile of inhibitory activities suggests that the main interacting enzyme form of eltrombopag might be the METTL3 subunit rather than the METTL14 subunit. In the case of the truncated form of the METTL3-14 complex, of which the crystal structure was reported, eltrombopag maintained its full inhibitory activities with a 3.5-fold decreased IC_50_ value of 12.0 μM (Figure 5A). Therefore, it could be predicted that some part of the binding regions for eltrombopag might exist in the truncated form of the METTL3 monomer. 

Consequently, potential allosteric binding pockets were investigated in the METTL3 crystal structure (PDB: 5IL1 A chain) through computational cavity searching algorithms using Allosite [48] and Discovery Studio software (version 3.5). As a result, a new allosteric binding pocket—distinct from the SAM binding site—was simultaneously predicted by both of the independent programs (Figure 5B). Then, a molecular docking experiment was performed on eltrombopag at the predicted allosteric binding pocket using the CDOCKER protocol of the Discovery Studio software. As illustrated in Figure 5B,C, eltrombopag was successfully docked to the predicted binding pocket, displaying several interactions with amino acids in the region. The putative binding modes include the hydrogen bonding interactions between the backbone amide group of Asp499 and Cys500 residues, the carboxylate group of Asp453, and the carboxamide group of Gln 496 with the carboxylic acid, the phenolic alcohol, and the hydrazine of eltrombopag, respectively. Additionally, some van der Waals interactions with aromatic groups of eltrombopag were predicted with several hydrophobic amino acids, including Val452, Val485, and Val487. It might be speculated that the putative binding could be further tightened by more interactions with the missing domains from the full-length METTL3-14 complex, such as the zinc finger domain.

The attempt of the X-ray analysis to verify the predicted binding site of eltrombopag at METTL3 was unfortunately not feasible since it was hard to generate a crystal of eltrombopag with METTL3 due to the low water solubility of eltrombopag [49]. Therefore, the interactions of eltrombopag in the predicted binding site of METTL3-14 were indirectly investigated by evaluating the inhibitory activities of its derivatives, thereby disrupting the hydrogen bonding donors of eltrombopag, such as carboxylic acid or the phenol groups, which might be responsible moieties for the key interactions in docking results. Thus, compounds **14a** (removal of carboxylic acid), **14b** (carboxylate methyl ester), and **14d** (removal of phenol group) were synthesized as Scheme S1 in the Supplementary Materials and evaluated for their enzyme inhibitory activities.

**Figure 6:**
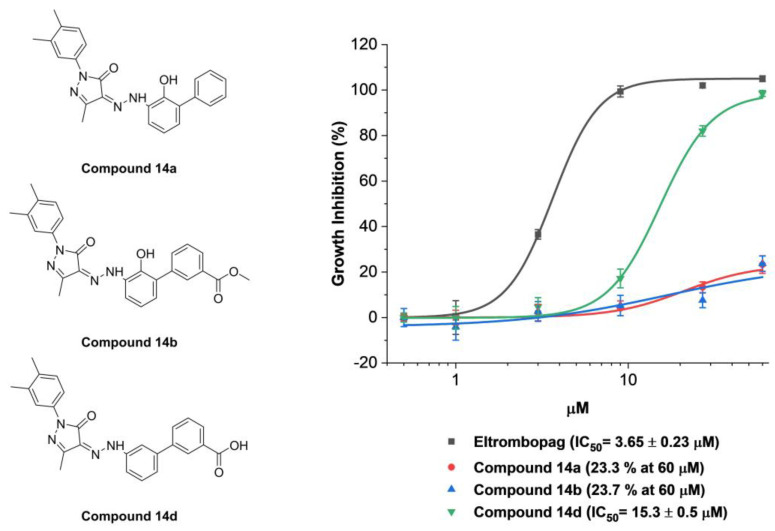
METTL3-14 enzymatic inhibitory activities of compounds **14a**, **14b**, and **14d** with structural modifications to disrupt the predicted hydrogen bonding interactions between METTL3-14 and eltrombopag.

As a result, compounds **14a**,**b** exhibited a significant loss of METTL3-14 inhibitory activities with only 23.3 and 23.7% inhibitions even at 60 μM, respectively, indicating that the acidic proton of the carboxylic acid moiety of eltrombopag is essential for maintaining the inhibitory activity (Figure 6). In addition, the importance of phenolic alcohol in maintaining the inhibitory activity of eltrombopag was also confirmed and observed in the 4-fold decreased inhibitory activity of compound **14d** with an IC_50_ value of 15.3 μM. Collectively, these results, consistent with the reduced docking scores in the molecular docking experiments, indirectly support the predicted binding mode of eltrombopag in a complex with METTL3.

### 2.3. Cellular Activity Evaluation of Eltrombopag on Acute Myeloid Leukemia Cell Lines

**Figure 7:**
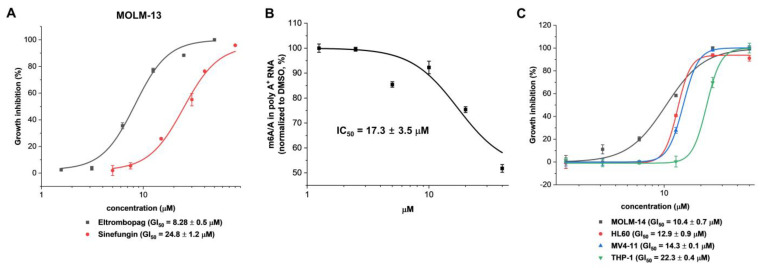
Anti-proliferative activity and cellular activity of eltrombopag on AML cell lines. (**A**) Anti-proliferative activity of eltrombopag against the MOLM-13 cell line. A global methyltransferase inhibitor, Sinefungin (**1**), was evaluated as a positive control in MOLM-13 cell line with a GI_50_ value of 24.8 μM. (Gilteritinib, an FLT3 targeted anti-AML drug, showed a GI_50_ value of 16.6 nM in Figure S3). (**B**) Dose-response curve of m^6^A/A reduction on poly-A^+^-enriched mRNA after 24 h of MOLM-13 treatment with eltrombopag. (**C**) Anti-proliferative activities of eltrombopag against AML cell lines including MOLM-14, HL60, MV4-11, and THP-1 cell lines.

Based on the close relationship of METTL3-14 for the proliferation of AML, the cellular level of anti-leukemic potential associated with the inhibition of METTL3-14 was investigated by the anti-proliferative activity of eltrombopag in MOLM-13 as an AML cell line closely related with METTL3 for its growth [32,33]. As shown in Figure 7A, eltrombopag exhibited the growth inhibition of the MOLM-13 cell line with a GI_50_ value of 8.28 μM. In addition, dose-dependent inhibition of m^6^A levels on poly-A^+^-enriched mRNA was also confirmed in the MOLM-13 cell line after 24 h eltrombopag treatment (Figure 7B), indicating that eltrombopag exhibited anti-leukemic effects on the MOLM-13 cell line by a correlated reduction of the m^6^A levels through the inhibition of METTL3-14. The maximum m^6^A inhibition by eltrombopag in MOLM-13 cells was shown to be 50% at 40 μM, which was similar to those of the shMETTL3 treated case (Figure S4) and the previously reported data (60–70%) with UZH1a [37]. Moreover, eltrombopag also showed the antiproliferative effects against other AML cell lines, including MOLM-14, HL60, MV4-11, and THP-1, with a range of GI_50_ values from 10~22 μM (Figure 7C).

The Steidl group has previously reported that eltrombopag displayed anti-proliferative activity, inducing the differentiation of human acute myeloid cells, which have an independent biological activity at the original target, TPO-R, without presenting a clear mechanism of eltrombopag for anti-leukemic effects [50]. In the present study, we have demonstrated that the mechanism of action of eltrombopag for its anti-leukemic effects would be direct inhibition of METTL3-14 and a consequent decrease of the m^6^A levels of mRNA. In addition, the in–vivo anti-leukemic effect of eltrombopag, through prolonging the survival of a mouse model transplanted with an AML cell line, has been reported in the abovementioned paper [50]. Therefore, eltrombopag might be a starting point for the development of a novel mechanism-based drug candidate for the treatment of acute myeloid leukemia through a further optimization study of the chemical structure of eltrombopag based on its binding mode in METTL3-14.

**Figure 8:**
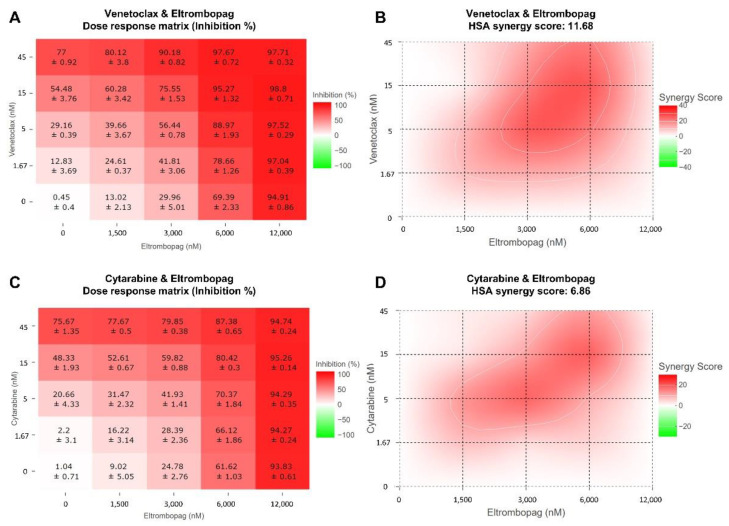
Synergistic effect of the combination treatment of eltrombopag and current AML drugs, venetoclax and cytarabine, on MOLM-13. (**A**) Dose-response matrix for the venetoclax/eltrombopag combination in MOLM-13 cell line. (**B**) 2D synergy maps and calculated HSA synergy scores for the venetoclax/eltrombopag combinations using Synergyfinder software. (**C**) Dose-response matrix for the cytarabine/eltrombopag combination in MOLM-13 cell line. (**D**) 2D synergy maps and calculated HSA synergy scores for the cytarabine/eltrombopag combinations using Synergyfinder software. Red and green areas represent synergy and antagonism, respectively. All experiments were repeated at least three times.

To investigate the applicability of eltrombopag for combination therapy with current AML drugs, we evaluated the antiproliferative activity of combination treatments of eltrombopag and current AML drugs, including venetoclax, cytarabine, gilteritinib, and sorafenib, against MOLM-13. The synergy scores for each combination were calculated using the Highest Single Agent (HSA) model in the Synergyfinder software [51]. As a result, a clear synergistic inhibitory effect of the venetoclax/eltrombopag combination was determined with an average HSA synergy score of 11.68 and a peak value of 26.37 (Figure 8A,B). In addition, this synergistic effect of the venetoclax/eltrombopag combination was further confirmed from the analysis with the Zero Interaction Potency (ZIP), Loewe additivity model, and Bliss independence models in the Synergyfinder software [51]. In the case of the cytarabine/eltrombopag combination, a relatively weak synergistic effect was observed with an average HSA synergy score of 6.86 (Figure 8C,D), which was consistent with the previously reported result that eltrombopag enhanced the anticancer effect of cytarabine [52]. However, the treatments with the combination of eltrombopag with gilteritinib or sorafenib didn’t display a significant synergistic effect (Figure S5). Collectively, the results indicated that eltrombopag could have a potential for use in combination with current AML drugs, including venetoclax and cytarabine, for the treatment of AML.

In clinical studies of eltrombopag, although monotherapy treatment didn’t show any safety concerns, increased progression to acute myeloid leukemia was observed in the combination treatment with azacitidine compared with the treatment of azacitidine alone [53,54]. Therefore, attention to various safety concerns should be drawn when attempting a combination therapy of drugs with eltrombopag.

### 2.4. Identification of Anti-Leukemia Potential of Eltrombopag at Molecular Level

**Figure 9:**
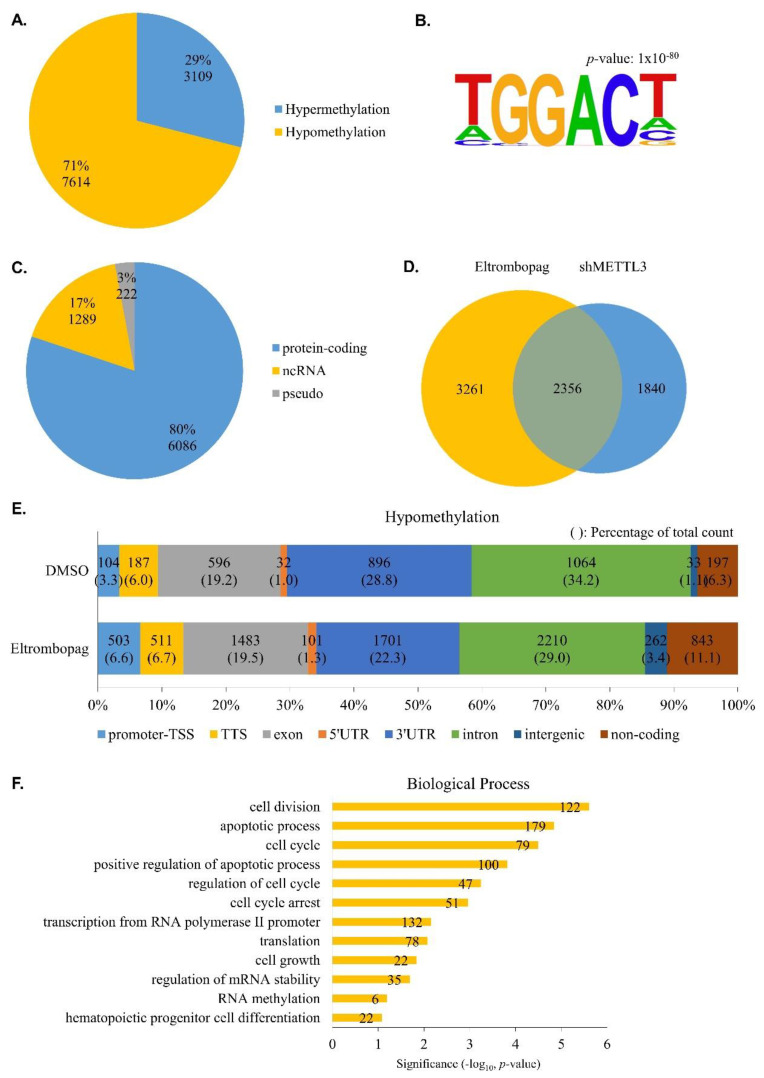
Eltrombopag reduces m^6^A levels in AML cells. (**A**) Distribution of significantly differentiated m6A methylated peaks detected in MOLM-13 cell line with 25 μM of eltrombopag. If diff.log2.fc is larger than 0 it is indicated as hypermethylation (blue), and if smaller than 0 it is indicated as hypomethylation (yellow). (**B**) Top consensus motif in eltrombopag-treated differentiated m^6^A methylated peaks with HOMER. GGAC, a subset of the m^6^A common motif, was detected. (**C**) Distribution of hypomethylated gene identity. Protein-coding, non-coding RNA (ncRNA), and pseudogenes (pseudo) are shown in blue, yellow, and gray, respectively. (**D**) Common and unique hypomethylated peaks in eltrombopag-treated or shMETTL3-treated MOLM-13 cell lines. A proportional Venn diagram was drawn with VennDiagram. (**E**) Peak annotation of DMSO or eltrombopag-treated hypomethylation with HOMER. TSS, transcription start site (from −1 kb to +100 bp); TTS, transcription termination site (from −100 bp to +1 kb); UTR, untranslated region. (**F**) Gene ontology enrichment analysis of eltrombopag-treated hypomethylation genes. The number of genes associated with the GO term is shown at the end of the bar.

To determine whether eltrombopag has anti-leukemia potential on a molecular level, we performed N^6^-methyladenosine-sequencing (m^6^A-seq) using the eltrombopag-treated MOLM-13 cell line and analyzed the differential m^6^A methylation sites in the transcriptome. As shown in Figure 9A and Table S1, we identified 10,723 differential m^6^A methylation sites, and 71% of them were hypomethylated, similarly to the previously reported METTL3 inhibitor STM2457 treatment [39]. Additionally, we found that about 30% of hypermethylation was also detected when treated with eltrombopag or STM2457. To find out whether this hypermethylation is specifically caused by chemical METTL3 inhibitors, we obtained m^6^A individual-nucleotide-resolution cross-linking and immunoprecipitation (miCLIP) sequencing results for the METTL3-deficient MOLM-13 cell line from the Gene Expression Omnibus (GEO) database [32] and re-analyzed the differential m^6^A methylation sites. As a result, hypomethylation was predominant in the METTL3 knockdown cells, but hypermethylation was also observed (Figure S6, Table S2). Our observations regarding hypermethylation upon METTL3 inhibition suggest that it might be considered in the development of anti-AML therapies. 

M^6^A RNA methylation tends to occur in the RRACH consensus motif [2,4]. To investigate the specificity of eltrombopag-regulated m^6^A methylation, we analyzed motifs in differentially methylated peaks. Figure 9B shows that GGAC, a subset of the m^6^A common motif, is highly enriched in its differential methylation sites. Since eltrombopag acts as a METTL3 inhibitor, we focused on the hypomethylation genes in subsequent analyses. We further analyzed the types of hypomethylated genes, and, as a result, most of them were protein-coding (~80%), noncoding-RNA (ncRNA), and pseudogenes accounting for 17% and 3%, respectively (Figure 9C).

Next, we compared hypomethylated genes in eltrombopag-treated or METTL3 knockdown cells. As shown in Figure 9D, a significant number of them overlapped, suggesting that eltrombopag leads to gene hypomethylation through the METTL3 inhibitory effect. In addition to the overlapping regions, there were eltrombopag-specific hypomethylated genes, which may be due to eltrombopag not targeting METTL3 alone but inhibiting the roles of other components of the METTL3-14 complex. To further examine the m^6^A peak distribution affected by eltrombopag, we performed a peak annotation analysis. As a result, hypomethylation in the 3′UTR and intron, in which the m^6^A peak is widely distributed, was significantly decreased during eltrombopag treatment, whereas it was increased in the promoter-TSS and non-coding region (Figure 9E, Table S1).

Finally, we applied gene ontology enrichment analysis to an in-depth study of the biological process of differentially eltrombopag-treated hypomethylated genes. As shown in Figure 9F, hypomethylated genes were highly enriched in apoptotic processes, cell cycle and growth, and hematopoietic progenitor cell differentiation events, suggesting that eltrombopag might be involved in the physiological processes of AML cells. A recent study has shown that the N^6^-methyltransferase complex, including METTL3-14, is recruited near the promoter and promotes RNA polymerase II pausing, leading to gene regulation [55]. The eltrombopag-treated m^6^A peak distribution at the promoter-TSS and transcription-related events shown in the GO analysis suggest that eltrombopag may affect gene expression through the regulation of m^6^A RNA methylation around promoter-TSS (Figure 9E, Table S3). Taken together, eltrombopag reduced m^6^A levels in the MOLM-13 transcriptome, indicating the anti-leukemia potential of eltrombopag on a molecular level.

## 3. Materials and Methods

### 3.1. General Methods for Chemistry 

All reagents and solvents were obtained from commercial sources and used without further purification. Eltrombopag (**1**) was purchased from Axonmedchem (SB 497115) (Groningen, The Netherlands). The ^1^H and ^13^C Nuclear magnetic resonance (NMR) spectra were recorded with a JEOL JNM-ECX 400p spectrometer at 400 MHz and 101 MHz, respectively. All spectra were taken using CDCl_3_ and dimethyl sulfoxide (DMSO)-d_6_. Mass spectroscopy was performed on a BEH C18 column (1.7 μM, 2.1 mm × 50 mm; Waters) and maintained at 40 °C during separation under isocratic conditions (mobile phase A: mobile phase B = 20:80) by using a Waters ACQUITY ultraperformance liquid chromatograph coupled to a triple quadrupole mass spectrometer (Micromass Quattro Micro, Waters, Milford, MA, USA). The mobile phase was as follows: A, water (LC-MS grade) with 0.1% formic acid (*v*/*v*); and B, CH3CN (LC-MS grade) with 0.1% formic acid (*v*/*v*); flow rate, 0.2 mL/min. Synthetic procedures and characterization data for all the synthesized compounds are provided in the Supplementary Materials.

### 3.2. Cloning, Expression and Purification of METTL3 and METTL14

For recombinant protein production, genes encoding the full-length and MTase domain of human METTL3 (residues 369 to 580) or METTL14 (residues 106 to 396) were cloned between the XbaI and NotI sites in the pVL1393 baculovirus transfer vector (BD Biosciences, NJ, USA) with a thrombin-cleavable decahistidine (10× His) tag at the C-terminus and N-terminus. The plasmids were transfected into Spodoptera frugiperda (Sf9) cells using BestBac 2.0 linearized baculovirus DNA (Expression Systems, Davis, CA, USA) and Cellfectin II transfection reagent (Gibco, Waltham, MA, USA). The METTL3-METTL14 complex was obtained by co-infection of Trichoplusia ni (Hi5) cells with two recombinant baculoviruses. The transgenic cells were cultured for 72 h at 28 °C and harvested by centrifugation at 14,000×  *g* for 10 min at 4  °C. 

The purification of the human METTL3 and METTL14 complex was performed with slight modifications [22]. Briefly, cells were re-suspended and broken using a sonicator (6 cycles of 10 s on/10 s off, 40% amplitude, Branson, CT, USA) in a lysis buffer containing 20 mM Tris-HCl pH 8.0, 200 mM NaCl, 10 μg/mL DNase I and 0.1 mM phenylmethylsulfonyl fluoride (PMSF). Cell debris was removed by ultracentrifugation at 240,000×  *g* for 1 h at 4  °C, and the supernatant was loaded onto Ni-NTA affinity resin (Goldbio, MO, USA). The resin was washed with 20 column volumes of lysis buffer containing 50 mM imidazole. The protein was eluted with a 100-, 300-, and 500-mM imidazole gradient. After cleavage by thrombin to remove the histidine tag, the protein was further purified by HiTrap Q (GE Healthcare, Chicago, IL, USA) anion exchange chromatography and Superdex 200 Increase (GE Healthcare) gel filtration chromatography in a buffer containing 25 mM Tris-HCl pH 8.0, 150 mM NaCl, and 3 mM DL-Dithiothreitol (DTT, Goldbio). Fractions containing the complex protein were pooled and concentrated to an amount of 1 mg/mL for functional assays. All purification steps were performed on ice or at 4 °C.

### 3.3. METTL3-14 Enzyme-Based Bioluminescence Assay (Screening Assay)

An enzymatic bioluminescence assay was established to identify the METTL3-14 inhibitor through screening. During the screening, all enzymatic reactions were performed in 96-well plates using a reaction buffer (20 mM Tris pH 7.5, 1 mM DTT, 0.01% Triton X-100, and 40 U of RNaseOUT (Invitrogen, Waltham, MA, USA) with a final reaction volume of 20 μL. In total, a final concentration of 30nM of METTL3-14 was incubated with 300 nM final concentration of SAM, 300 nM final concentration of RNA substrates (5′-ACGAGUCCUGGACUGAAACGGACUUGU-3′), and serially diluted compounds at 23 °C for 1 h. Furthermore, 5 μL of 5% trifluoroacetic acid was added to each well to stop the enzymatic reaction. After a 10-min incubation period, the reaction product, SAH, was converted to ATP as a substrate of luciferase using the MTase-GloTM Methyltransferase Assay kit (Promega, Madison, WI, USA). METTL3-14 activity was assessed by measuring the luminescence using a Victor multilabel reader (PerkinElmer, Waltham, MA, USA). The IC_50_ values were calculated by a nonlinear regression analysis using OriginPro 9.1 software (OriginLab).

To confirm the mechanism of action of eltrombopag in the METTL3-14 complex, 30 nM METTL3-14 and serially diluted eltrombopag were reacted with various concentrations of SAM or RNA substrate and saturating concentrations of the other substrate (3 μM SAM or 2 μM RNA substrate) for 20 min. To evaluate the inhibitory activity in a single form of METTL3 and METTL14, 1 μM of METTL3 and 300 nM of METTL14 were incubated with 1 μM of SAM, 500 nM of RNA substrates, and serially diluted eltrombopag for 5 h. To measure the inhibitory activity in the truncated form of the METTL3-14 complex, 1 μM of the truncated METTL3-14 complex was reacted with 1 μM of SAM, 500 nM of RNA substrates, and serially diluted eltrombopag for 5 h.

### 3.4. METTL3-14 Enzyme-Based Bioluminescence Assay (False Positive Response Experiment)

To determine whether hit compounds are false positives that inhibit the coupled enzyme reaction processes converting SAH to ATP, 30 nM of METTL3-14, 300 nM of SAM, and 300 nM of RNA substrates were incubated at 23 °C for 1 h without a hit compound. After incubation, 5 μL of 5% trifluoroacetic acid was added to each well to stop the enzymatic reaction. Then, the hit compound was added to the reaction mixture at a concentration of 25 μM before the product SAH was converted to ATP. Finally, the reaction product, SAH, was converted to ATP as a substrate of luciferase using the MTase-GloTM Methyltransferase Assay kit (Promega). METTL3-14 activity was assessed by measuring the luminescence using a Victor multilabel reader (PerkinElmer). If it shows the inhibitory activity in a false positive response experiment, this hit compound is a false positive affecting the coupled enzyme system. 

To investigate whether the hit compound was a false positive, thereby causing colloidal aggregation, a hit compound diluted in enzyme assay buffer was centrifuged at 15,000× *g* for 10 min at 4 °C. After centrifugation, the supernatant was used for the METTL3-14 enzyme-based bioluminescence assay as described above. According to this method, if it is not possible to identify the inhibitory activity in a false positive centrifugation test, the hit compound is a false positive that will lead to colloidal aggregation. 

### 3.5. Mass Spectrometry Based METTL3-14 Enzyme Based Assay 

The 30 nM of METTL3-14 was incubated with 300 nM of SAM, 300 nM of RNA substrates, and serially diluted compounds at 23 °C for 1 h as described above. The enzymatic reaction was stopped by heating at 80 °C for 10 min and centrifuged at 15,000× *g* for 10 min. Then, the reacted RNA in the supernatant was digested to nucleosides by using 2 units of nuclease P1 (NEB, Ipswich, MA, USA) and dephosphorylated by using 1 unit of alkaline phosphatase (NEB) at 37 °C for 2 h.

The produced nucleosides were detected by reversed-phase high-performance liquid chromatography on an ACQUITY BEH C18 column (1.7 μM, 2.1 mm × 50 mm; Waters) coupled with mass spectrometry detection using an EVOQ Elite ER LC-TQ (Bruker, Billerica, MA, USA). The adenosine and m^6^A were quantified using an MRM transition as follows: *m*/*z* = 267.9 -> 136.1 and *m*/*z* = 282.1 -> 150.1, respectively. (Retention time 5 min). The m^6^A/A nucleoside ratio of the eltrombopag-treated sample was normalized to the corresponding value of the DMSO treated negative control. The IC_50_ values were calculated by a nonlinear regression analysis using OriginPro 9.1 software (OriginLab).

### 3.6. Surface Plasmon Resonance

The interaction between eltrombopag and the METTL3-14 complex was explored using a Reichert SR7000DC instrument optical biosensor (Reichert Technologies, Depew, NY, USA) equipped with a carboxymethyl dextran sensor chip (part no. 13206066, Reichert Technologies). To activate the sensor chip, free carboxyl groups on the surface were modified by injecting a mixture of 0.1 M of 1-ethyl-3-(3-dimethylaminopropyl) carbodiimide hydrochloride and 0.05 M of N-hydroxysuccinimide at a flow rate of 10 μL/min to generate a reactive succinimide ester surface. Then, the METTL3-14 complex (9.5 μg/mL; prepared in 10 mM sodium acetate buffer, pH 4.5) was coupled to the sensor via free amine coupling to the immobilized succinimide and followed by the quenching of the remaining activated succinimide ester with 1 M of ethanolamine, pH 8.5. The eltrombopag was diluted in PBS that was supplemented with 0.005% Tween, thereby maintaining a final 5% DMSO concentration. The binding experiments were performed using a flow rate of 30 μL/min with an association time of 5 min and a dissociation time of 7 min. The regeneration of the surfaces was performed, when necessary, by 10 mM of NaOH. The Langmuir model of the Scrubber2 software was used to determine the equilibrium dissociation constant and kinetic dissociation and association constants. 

### 3.7. Selectivity Profiling 

The selectivity profile of eltrombopag was investigated by testing the level of inhibition in a methyltransferase panel. The inhibition of 7 RNA methyltransferase was tested at Reaction Biology (Malvern, PA, USA) using a gold standard radioisotope-based MT assay (MT HotSpot^TM^) with 10 μM of eltrombopag in duplicate. SAH and chaetocin were used as positive controls in the methyltransferase panel screening.

### 3.8. Allosteric Binding Pocket Prediction and Molecular Docking 

The potential allosteric binding pocket in METTL3 was independently predicted based on the METTL3 crystal structure (PDB ID: 5IL1 A chain) obtained from the Protein Data Bank via the “From Receptor Cavities” protocol in the Discovery Studio 3.5 software and Allosite 2.0 [48], which detects allosteric sites based on a structure-based machine learning method. After prediction, the protein preparation process was carried out using the “Prepare Protein” wizard included in Discovery Studio and a radius of 15 Å around the predicted allosteric binding pocket was set as a binding site. For the molecular docking, ligands applied with a CHARMM force field were docked into the above-predicted allosteric binding pocket in METTL3 using the CDOCKER protocol in Discovery Studio.

### 3.9. Cell Culture and shMETTL3 Knockdown

The MOLM-13, MOLM-14, and MV4-11 cell lines were supplied by the Leibniz Institute DSMZ-German Collection of Microorganisms and Cell Cultures GmbH (DSMZ) (Braunschweig, Germany). The HL60 and THP-1 cell lines were supplied by the Korea Cell Line Bank (KCLB) (Seoul, Korea). The MOLM-13, MOLM-14, HL60, MV4-11, and THP-1 cells were cultured in RPMI 1640 medium (HyClone, Logan, UT, USA) with 10% fetal bovine serum (Gibco) and 1% penicillin/streptomycin in 5% CO_2_ at 37 °C in a humidified incubator. METTL3 shRNA was produced by transfection of shRNA plasmid DNA (Open Biosystems), psPAX2 (packaging plasmid), and pMD2.G (envelope plasmid) with polyethyleneimine (PEI) reagent into HEK293T cells. After 24 h, the media were freshly replaced, and 48 h after transfection the supernatant was passed through a 0.45 μm filter to obtain a viral supernatant. The viral supernatant was transfected into MOLM-13 cells using a polybrene reagent.

### 3.10. Anti-Proliferative Assay Protocol

The MOLM-13, MOLM-14, HL60, MV4-11, and THP-1 cells were seeded in white 96-well clear bottomed plates with 100 μL of medium (5000 cells/well). The plated cells were treated with serially diluted compounds and incubated for 72 h at 37 °C. After incubation, 10 μL of EZ-cytox kit reagent from the EZ-cytox cell viability assay kit (DaeiLab, Seoul, Korea) was added to each well and then incubated for 3 h at 37 °C. The absorbance of metabolically active cells was measured at a wavelength of 450 nm using a Victor multilabel reader (PerkinElmer). The GI_50_ values were calculated by nonlinear regression analysis using OriginPro 9.1 software (OriginLab).

### 3.11. Combinatorial Analysis of AML Drugs with Eltrombopag

The MOLM-13 cells were seeded in white 96-well clear bottomed plates with 50 μL of medium (5000 cells/well). The plated cells were treated with serially diluted eltrombopag and current AML drugs, including venetoclax, cytarabine, gilteritinib, and sorafenib, to a final volume of 100 μL and incubated for 72 h at 37 °C. After incubation, 10 μL of EZ-cytox kit reagent from the EZ-cytox cell viability assay kit (DaeiLab) was added to each well and then incubated for 3 h at 37 °C. The absorbance of metabolically active cells was measured at a wavelength of 450 nm using a Victor multilabel reader (PerkinElmer). The synergy scores of each combination were quantified by the HAS model, ZIP model, Loewe additivity model, and Bliss independence models using Synergyfinder software [51]. Positive or negative synergy scores obtained through these models represent synergy and antagonism, respectively.

### 3.12. Quantitative Analysis of m^6^A Level by Mass Spectrometry

The MOLM-13 cells were seeded in 10 cm^2^ dishes at a density of 1.5 × 10^6^ cells/mL with 10 mL of complete medium. After 24 h, the plated cells were treated with the vehicle (DMSO) or serial dilutions of eltrombopag and incubated for 24 h. Following incubation in the cell culture incubator, total RNA was extracted using TRIsure^TM^ (Bioline, Brisbane, Australia) according to the manufacturer’s instructions. Then, poly-A^+^-enriched mRNA was purified from the extracted total RNA using the Magnosphere^TM^ ultrapure mRNA purification kit (Takara, Shiga, Japan). One microgram of mRNA was digested to the nucleosides using 2 units of nuclease P1 (NEB) and dephosphorylated using 1 unit of alkaline phosphatase (NEB) at 37 °C for 2 h.

The produced nucleosides were detected by reversed-phase high-performance liquid chromatography on an ACQUITY BEH C18 column (1.7 μm, 2.1 mm × 50 mm; Waters) coupled with mass spectrometry detection using an EVOQ Elite ER LC-TQ (Bruker). The adenosine and m^6^A were quantified using MRM transition as follows: *m*/*z* = 267.9 -> 136.1 and *m*/*z* = 282.1 -> 150.1, respectively. (Retention time 5 min). The m^6^A/A nucleoside ratio of the eltrombopag-treated sample was normalized to the corresponding value of the DMSO treated negative control. The inhibition curves were plotted using OriginPro 9.1 software (OriginLab) and fitted using nonlinear regression analysis.

### 3.13. N^6^-Methyladenosine-Sequencing (m^6^A-seq) and Sequencing Data Analysis

mRNA was purified using a GenElute™ mRNA Miniprep Kit (SIGMA, MRN10-1KT, St. Louis, MO, USA) according to the manufacturer’s protocol. Briefly, the total RNA was mixed with 2× Binding Solution and oligo(dT) beads, vortexed, and left for 10 min at room temperature. The beads:mRNA complex was passed through a GenElute spin filter/collection tube and washed twice with Wash Solution. The mRNA was eluted in an Elution Solution incubated at 70 °C.

m^6^A-seq was performed using the EpiMark^®^ N^6^-Methyladenosine Enrichment Kit (NEB, E1610S). Briefly, 25 μL of Dynabeads™ Protein G (Invitrogen, 10004D) and 1 μL of N^6^-Methyladenosine Antibody were incubated in a 4 °C rotator for 2 h. The purified mRNA was fragmented at 95 °C for 10 min in a fragmentation buffer (100 mM Tris (pH 8.0), 8 mM MgCl_2_) with spike-in control RNA (m^6^A and unmodified, 0.1 fmol of each RNA). The fragmented RNA was concentrated with RNA Clean & Concentrator™-5 (ZYMO RESEARCH, R1014, Irvine, CA, USA) and then 5% was saved as the input. The remaining 95% of the fragmented RNA was added to the beads/antibody complex. RNA was eluted in Buffer RLT (QIAGEN, 79216, Hilden, Germany), followed by ethanol precipitation and was used for sequencing library construction. All sequencing experiments were performed in triplicate via Novaseq. Adapter trimming and QC were performed using Trim Galore. Reads were mapped to hg38 by HISAT2 [56], and exomePeak [57] and HOMER [58] were used to analyze the differential methylation peaks, motifs, and gene annotations.

## 4. Conclusions

In this study, we reported on the use of eltrombopag as a METTL3-14 allosteric inhibitor that was identified through the screening of a drug library from the Korea Chemical Bank, and its mechanism of action was determined by various biochemical analyses. Eltrombopag exhibited selective inhibitory activity against the METTL3-14 complex with an IC_50_ value of 3.65 μM, and its direct binding to the enzyme complex was confirmed by the analysis in surface plasmon resonance experiments. In addition, exploring the mode of enzyme inhibitory mechanisms revealed that eltrombopag acts as a noncompetitive inhibitor interacting at a putative allosteric binding site predicted by molecular docking studies and the main enzyme of its interaction was experimentally determined as the METTL3 subunit rather than the METTL14 subunit. On a cellular level, eltrombopag showed anti-leukemic activity in the relevant AML cell line, MOLM-13, in correlation with the reduction of m^6^A levels. Molecular mechanism studies of eltrombopag using m^6^A-seq analysis provided further evidence of its cellular function by determining the hypomethylation of leukemogenic genes in eltrombopag-treated MOLM-13 cells and the overlapping of the pattern with those of the METTL3-knockdown MOLM-13 cells. Taken together, in this study, eltrombopag was identified as a METTL3-14 allosteric inhibitor with anti-leukemic activity against AML, which could provide a potential opportunity for the development of new drug candidates for AML via further optimization of the structure of eltrombopag at the binding site.

## Figures and Tables

**Table 1:** Methyltransferase selectivity profile of eltrombopag at 10 μM.

Methyltransferase	Remaining Activity (%) ^1^	Control IC_50_ (μM) ^2^	Control Compound
DOT1L	90.0 ± 2.8	0.217	Chaetocin
G9a	99.0 ± 3.3	0.762	SAH
MLL4 complex	70.9 ± 2.4	5.09	SAH
PRDM9	130 ± 4	4.16	SAH
PRMT1	96.1 ± 3.9	0.637	SAH
SETD2	97.3 ± 4.1	10.8	SAH
SMYD3	98.7 ± 5.7	35.3	SAH

^1^ The remaining activity is the percentage of enzymatic activity in the presence of 10 μM eltrombopag to the buffer containing DMSO. ^2^ IC_50_ (50% inhibitory concentrations of activity) values were obtained based on the concentration−response curves.

## Data Availability

Publicly available datasets were analyzed in this study. The m^6^A-seq and RNA-seq data created in this study are openly available in the NCBI Sequence Read Archive at BioProject accession number PRJNA807459.

## Supplementary Materials

The following supporting information can be downloaded at: https://www.mdpi.com/article/10.3390/ph15040440/s1, Scheme S1: Reagents and conditions: (a) Sodium acetate, AcOH, reflux, 24 h; (b) R^1^-PhB(OH)_2_, Pd(PPh_3_)_4_, K_2_CO_3_, THF/water/EtOH, 60 °C, 18 h; (c) Pd/c, THF/MeOH, rt, 3 h; (d) NaNO_2_, NaHCO_3_, MeOH, rt, 3 h; (e) KOH, MeOH, water, 60 °C, 18 h; Figure S1: Kinetic parameter determination of the METTL3-14 complex by using screening assay system, Figure S2: Confirmation of false-positive potential, Figure S3: The anti-proliferative activity of gilteritinib as positive control was confirmed a GI_50_ value of 16.6 nM in MOLM-13. Figure S4: m^6^A levels on poly-A+-enriched mRNA after 24 h of eltrombopag treatment (40 μM) or 4 days after post-transduction of siRNA in MOLM-13, Figure S5: Analysis of combination treatment of AML drugs, gilteritinib and sorafenib, with eltrombopag in MOLM-13, Figure S6: Distribution of significantly differential m^6^A methylated peaks detected in shMETTL3-treated Molm-13 cells, Figure S7: NMR spectrum of compound **14a**, Figure S8: NMR spectrum of compound **14b**, Figure S9: NMR spectrum of compound **14d**, Table S1: Differential m^6^A methylation sites and peak annotation in Eltrombopag-treated Molm-13 cells, Table S2: Differential m^6^A methylation sites in shMETTL3-treated Molm-13 cells, Table S3: GO enrichment analysis of differentially m^6^A methylated genes.
