# Supplementary material for: Eltrombopag as an Allosteric Inhibitor of the METTL3-14 Complex Affecting the m6A Methylation of RNA in Acute Myeloid Leukemia Cells
Source: Pharmaceuticals (Basel). 2022 Apr 1;15(4):440. doi: 10.3390/ph15040440 (PMC9032718; doi:10.3390/ph15040440)

Supplementary Materials

Eltrombopag as an Allosteric Inhibitor of the METTL3-14 Complex Affecting the m⁶A Methylation of RNA in Acute Myeloid Leukemia Cells

Je-Heon Lee †, Namjeong Choi †, Subin Kim, Mi Sun Jin *, Haihong Shen * and Yong-Chul Kim *

School of Life Sciences, Gwangju Institute of Science and Technology (GIST), Gwangju 61005, Korea; jhl@gm.gist.ac.kr (J.-H.L.); njchoi@gist.ac.kr (N.C.); ksb1201@gm.gist.ac.kr (S.K.)

* Correspondence: misunjin@gist.ac.kr (M.S.J.); haihongshen@gist.ac.kr (H.S.); yongchul@gist.ac.kr (Y.-C.K.)

† These authors contributed equally to this work.

[Table of Contents]

I. Synthetic scheme, procedure and characterization ..….………………… S3

II. Supporting figures and Tables

Kinetic parameter determination of the METTL3-14 complex by using screening assay system (Figure S1) ………………………………………….………...…. S7

Confirmation of false-positive potential (Figure S2) …..………………... S8

The anti-proliferative activity of gilteritinib as positive control was confirmed a GI_{50} value of 16.6 nM in MOLM-13. (Figure S3) …………………………..... S9

m^6A levels on poly-A+-enriched mRNA after 24h of eltrombopag treatment (40 μM) or 4 days after post-transduction of shRNA in MOLM-13 (Figure S4) …. S10

Analysis of combination treatment of AML drugs, gilteritinib and sorafenib, with eltrombopag in MOLM-13 ……….. S11

Distribution of significantly differential m^6A methylated peaks detected in shMETTL3-treated Molm-13 cells. (Figure S5) ……...……..…………... S12

NMR spectrum of compound 14a (Figure S5) ……...…….……………... S13

NMR spectrum of compound 14b (Figure S6) ……...…….……………... S14

NMR spectrum of compound 14d (Figure S7) ……...…….……………... S15

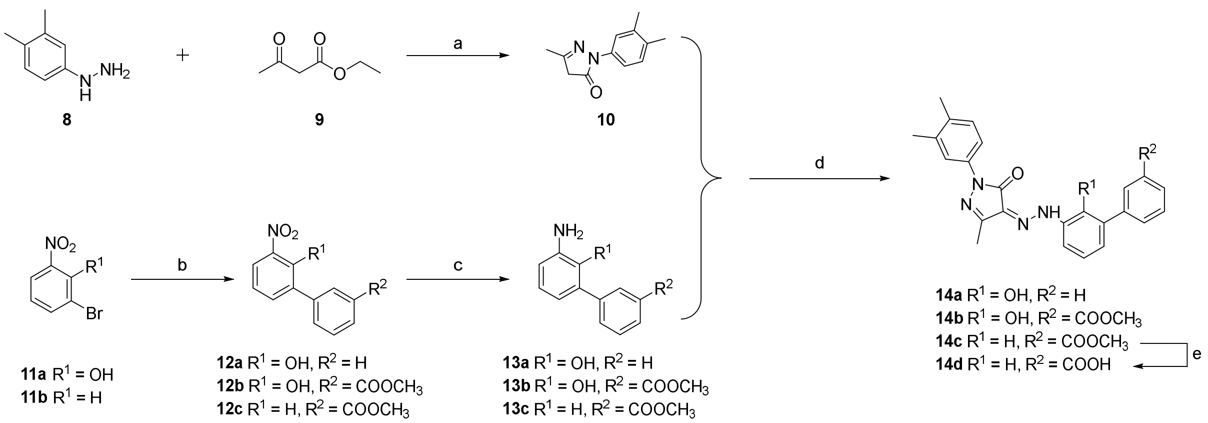

Scheme 1. Reagents and conditions: (**a**) Sodium acetate, AcOH, reflux, 24 h; (**b**) R^1-PhB$(OH)_2$, Pd$(PPh_3)_4$, K_2CO_3, THF/water/EtOH, 60 °C, 18 h; (**c**) Pd/c, THF/MeOH, rt, 3 h; (**d**) $NaNO_2$, $NaHCO_3$, MeOH, rt, 3 h; (**e**) KOH, MeOH, water, 60 °C, 18 h.

Firstly, the pyrazolone intermediate (**10**) was obtained by an acid-catalyzed condensation reaction between 3,4-dimthylphenylhydrazine (**8**) and ethyl acetoacetate (**9**) compounds. Secondly, aminobiphenyl intermediates (**13a-c**) were synthesized by palladium-catalyzed reduction of corresponding nitrobiphenyl derivatives (**12a-c**), which were prepared by Suzuki coupling reactions of starting compounds (**11a-b**) with the corresponding boronic acids. The synthesis of final products (**14a-c**) were accomplished by diazonium coupling reaction between intermediates **10** and **13a-c**. Another final compound (**14d**) was obtained from hydrolysis of the methyl ester compound (**14c**).

Synthetic procedure and characterization

2-(3,4-dimethylphenyl)-5-methyl-2,4-dihydro-3H-pyrazol-3-one (**10**). 3,4-Dimethylphenylhydrazine hydrochloride (**8**) (1.00 g, 5.79 mmol) was dissolved in AcOH (15 mL). Ethyl acetoacetate (**9**) (812 μL, 6.36 mmol) and sodium acetate (522 mg, 6.36 mmol) were added and the reaction was refluxed for 24 h with vigorous stirring. The solvent was evaporated under vacuum and the mixture was purified by silica gel column chromatography (DCM : MeOH = 100:1) to give **10** as a yellow oil (920 mg, 78.6% yield). ^{1}H NMR (400 MHz, $CDCl_3$) δ 7.59 - 7.64 (m, 1H), 7.56 (dd, J = 2.29, 8.24 Hz, 1H), 7.14 (d, J = 8.24 Hz, 1H), 3.39 (d, J = 0.69 Hz, 2H), 2.29 (s, 3H), 2.25 (s, 3H), 2.17 (s, 3H). MS (ESI): $[M + H]^+$ = 203.0.

3-nitro-[1,1'-biphenyl]-2-ol (**12a**). 2-Bromo-6-nitrophenol (**11a**) (500 mg, 2.29 mmol), phenylboronic acid

(363 mg, 2.98 mmol), Tetrakis(triphenylphosphine) palladium (0) (132 mg, 0.11 mmol), and K_2CO_3 (948 mg, 6.87 mmol) were dissolved in THF (30 mL), EtOH (2.5 mL), and H_2O (2.5 mL). The mixture was stirred at 65 °C for 18 h. After incubation, the reaction was filtered through Celite and the filtered residue was taken up in solution of saturated aq. NH4Cl and extracted with EtOAc. The combined extracts were dried over sodium sulfate, filtered and concentrated under vacuum. The residue was purified by silica gel column chromatography (Hx : EtOAc = 40:1) to give **12a** as a yellow solid (380 mg, 77.1% yield). ^{1}H NMR (400 MHz, $CDCl_3$) δ 11.15 (s, 1H), 8.15 (dd, *J* = 1.80, 8.70 Hz, 1H), 7.66 (dd, *J* = 1.60, 7.33 Hz, 1H), 7.53 - 7.61 (m, 2H), 7.39 - 7.52 (m, 3H), 7.07 (dd, *J* = 7.44, 8.59 Hz, 1H). MS (ESI): $[M - H]^-$ = 214.3.

methyl 2'-hydroxy-3'-nitro-[1,1'-biphenyl]-3-carboxylate (**12b**). **12b** was synthesized from **11a** and (3-(methoxycarbonyl)phenyl)boronic acid following the procedure described for **12a** as yellow solid (24.0% yield). ^{1}H NMR (400 MHz, $CDCl_3$) δ 11.14 (s, 1H), 8.23 (t, *J* = 1.83 Hz, 1H), 8.16 (dd, *J* = 1.83, 8.70 Hz, 1H), 8.07 - 8.11 (m, 1H), 7.77 (td, *J* = 1.80, 7.80 Hz, 1H), 7.66 (dd, *J* = 2.30, 6.90 Hz, 1H), 7.54 (t, *J* = 7.79 Hz, 1H), 7.03 - 7.13 (m, 1H), 3.95 (s, 3H). MS (ESI): $[M - H]^-$ = 272.3.

methyl 3'-nitro-[1,1'-biphenyl]-3-carboxylate (**12c**). **12c** was synthesized from **11b** and (3-(methoxycarbonyl)phenyl)boronic acid following the procedure described for **12a** as white solid (58.2% yield). ^{1}H NMR (400 MHz, $CDCl_3$) δ 8.48 (t, *J* = 1.83 Hz, 1H), 8.31 (s, 1H), 8.24 (ddd, *J* = 0.92, 2.29, 8.24 Hz, 1H), 8.11 (td, *J* = 1.37, 7.79 Hz, 1H), 7.96 (td, *J* = 1.43, 7.67 Hz, 1H), 7.82 (d, *J* = 7.79 Hz, 1H), 7.65 (t, *J* = 8.01 Hz, 1H), 7.59 (t, *J* = 7.79 Hz, 1H), 3.96 (s, 3H). MS (ESI): $[M + H]^+$ = 258.2.

3-amino-[1,1'-biphenyl]-2-ol (**13a**). **12a** (380 mg, 1.77 mmol) was dissolved in dried MeOH (10 mL) and THF (5 mL) with palladium on carbon (20.0 mg). The reaction mixutre was stirred at room temperature for 3 h under an atmosphere pressure of H_2. After reaction, the mixture was filtered through Celite and concentrated under vacuum. The mixture was purified by silica gel column chromatography (DCM : MeOH = 100:1) to give **13a** as a yellow solid (145 mg, 44.2% yield). ^{1}H NMR (400 MHz, $CDCl_3$) δ 7.47 - 7.55 (m, 4H), 7.37 - 7.45 (m, 1H), 6.85 (t, *J* = 7.67 Hz, 1H), 6.76 (dd, *J* = 1.60, 7.79 Hz, 1H), 6.70 (dd, *J* = 1.60, 7.56 Hz, 1H), 3.64 - 3.93 (m, 2H). MS (ESI): $[M + H]^+$ = 186.4.

methyl 3'-amino-2'-hydroxy-[1,1'-biphenyl]-3-carboxylate (**13b**). **13b** was synthesized from **12b**

following the procedure described for **13a** as yellow solid (46.0% yield). ^{1}H NMR (400 MHz, $CDCl_3$) δ 8.16 (t, J = 1.60 Hz, 1H), 8.04 (td, J = 1.40, 7.80 Hz, 1H), 7.69 (td, J = 1.40, 7.80 Hz, 1H), 7.55 (t, J = 7.80 Hz, 1H), 6.85 (t, J = 7.80 Hz, 1H), 6.78 (dd, J = 1.80, 7.80 Hz, 1H), 6.70 (dd, J = 1.83, 7.33 Hz, 1H), 3.97 - 4.82 (m, 2H), 3.92 (s, 3H). MS (ESI): $[M + H]^+$ = 244.4.

methyl 3'-amino-[1,1'-biphenyl]-3-carboxylate (**13c**). **13c** was synthesized from **12c** following the procedure described for **13a** as yellow solid (49.0% yield). ^{1}H NMR (400 MHz, $CDCl_3$) δ 8.22 - 8.27 (m, 1H), 7.99 (tdd, J = 0.92, 1.83, 7.79 Hz, 1H), 7.71 - 7.78 (m, 1H), 7.47 (t, J = 7.79 Hz, 1H), 7.23 (t, J = 7.80 Hz, 1H), 6.97 - 7.03 (m, 1H), 6.90 - 6.95 (m, 1H), 6.64 - 6.73 (m, 1H), 3.94 (s, 3H), 3.70 - 3.83 (m, 2H). MS (ESI): $[M + H]^+$ = 228.0.

(Z)-2-(3,4-dimethylphenyl)-4-(2-(2-hydroxy-[1,1'-biphenyl]-3-yl)hydrazineylidene)-5-methyl-2,4-dihydro-3H-pyrazol-3-one (**14a**). **13a** (50.0 mg, 0.27 mmol) was dissolved in MeOH (2.5 mL). 37% aq HCl (37.8 μl) was treated dropwise and the reaction was preincubated at room temperature for 20 min. Then, sodium nitrite (22.3 mg, 0.32 mmol) was added and the mixture was stirred at 5 °C for 1 h. After incubation, **10** (60.0 mg, 0.30 mmol) and sodium hydrogen carbonate (225 mg, 2.68 mmol) was added and the resulting mixture was stirred for additionally 1 h. The solid precipitate was collected by filtration, washed with MeOH, and dried in high vacuum to give **14a** as a yellow solid (72 mg, 66.9% yield). ^{1}H NMR (400 MHz, $CDCl_3$) δ 7.71 - 7.75 (m, 1H), 7.68 (dd, J = 2.29, 8.24 Hz, 1H), 7.47 - 7.56 (m, 5H), 7.39 - 7.47 (m, 1H), 6.97 - 7.22 (m, 4H), 2.36 (s, 3H), 2.27 (s, 3H), 2.31 (s, 3H). ^{13}C NMR (101 MHz, $CDCl_3$) δ 157.6, 147.5, 142.7, 137.2, 136.2, 135.8, 133.5, 129.9, 129.8, 129.2 (2), 129.0 (2), 128.5, 128.2, 127.0, 121.1, 119.8, 116.2, 115.3, 19.9, 19.3, 11.8. MS (ESI): $[M + H]^+$ = 399.1.

methyl (Z)-3'-(2-(1-(3,4-dimethylphenyl)-3-methyl-5-oxo-1,5-dihydro-4H-pyrazol-4-ylidene)hydrazineyl)-2'-hydroxy-[1,1'-biphenyl]-3-carboxylate (**14b**). **14b** was synthesized from **13b** following the procedure described for **14a** as yellow solid (86.1% yield). ^{1}H NMR (400 MHz, $CDCl_3$) δ 8.21 (t, J = 1.60 Hz, 1H), 8.05 (d, J = 7.79 Hz, 1H), 7.68 - 7.77 (m, 2H), 7.65 (dd, J = 1.83, 8.24 Hz, 1H), 7.53 (t, J = 7.80 Hz, 1H), 7.40 (dd, J = 1.37, 8.24 Hz, 1H), 7.10 - 7.20 (m, 2H), 7.05 (t, J = 7.80 Hz, 1H), 3.93 (s, 3H), 2.35 (s, 3H), 2.30 (s, 3H), 2.26 (s, 3H). ^{13}C NMR (101 MHz, $CDCl_3$) δ 166.7, 157.6, 147.2, 143.5, 137.2, 136.9, 135.6, 133.7, 133.6, 130.7, 130.2, 129.9, 129.4, 129.0, 128.9, 128.1, 127.7, 127.3, 121.1, 119.7, 116.2, 116.2, 52.2, 20.0,

19.3, 11.8. MS (ESI): $[M + H]^+$ = 457.6.

methyl (Z)-3'-(2-(1-(3,4-dimethylphenyl)-3-methyl-5-oxo-1,5-dihydro-4H-pyrazol-4-ylidene)hydrazineyl)-[1,1'-biphenyl]-3-carboxylate (**14c**). **14c** was synthesized from **13c** following the procedure described for **14a** as yellow solid (64.5% yield). ^{1}H NMR (400 MHz, DMSO-d_6) δ 8.25 (s, 1H), 8.00 (t, J = 7.33 Hz, 2H), 7.84 - 7.96 (m, 1H), 7.72 - 7.81 (m, 1H), 7.60 - 7.72 (m, 3H), 7.44 - 7.60 (m, 2H), 7.08 - 7.25 (m, 1H), 3.91 (s, 3H), 2.32 (s, 3H), 2.26 (s, 3H), 2.22 (s, 3H). MS (ESI): $[M + H]^+$ = 441.0.

(Z)-3'-(2-(1-(3,4-dimethylphenyl)-3-methyl-5-oxo-1,5-dihydro-4H-pyrazol-4-ylidene)hydrazineyl)-[1,1'-biphenyl]-3-carboxylic acid (**14d**). **14c** (53.0 mg, 0.12 mmol) was dissolved in MeOH (2 mL) and THF (2 mL). 20% aq. KOH (2 mL) was added dropwise and the reaction was stirred at 60 ºC for 18 h. The residue was taken up in solution of saturated aq. NH_4Cl and extracted with EtOAc. The combined extracts were dried over sodium sulfate, filtered and concentrated under vacuum. The mixture was purified by silica gel column chromatography (DCM : MeOH = 40:1) to give **14d** as a yellow solid (38.2 mg, 74.3% yield). ^{1}H NMR (400 MHz, DMSO-d_6) δ 8.26 (t, J = 1.83 Hz, 1H), 7.93 - 8.01 (m, 3H), 7.72 (d, J = 1.83 Hz, 1H), 7.50 - 7.70 (m, 5H), 7.20 (d, J = 8.24 Hz, 1H), 2.31 (s, 3H), 2.27 (s, 3H), 2.23 (s, 3H). ^{13}C NMR (101 MHz, DMSO-d_6) δ 167.1, 156.3, 148.1, 142.1, 140.5, 139.6, 136.7, 135.7, 132.7, 131.6, 131.0, 130.3, 129.7, 129.3, 128.6, 128.1, 127.3, 123.8, 118.8, 115.2, 115.1, 114.8, 19.6, 18.7, 11.5. MS (ESI): $[M + H]^+$ = 427.1.

Figure S1. Kinetic parameter determination of the METTL3-14 complex by using screening assay system. (**A)** Michaelis constant (K_m value) of RNA. (**B**) Michaelis constant (K_m value) of SAM.

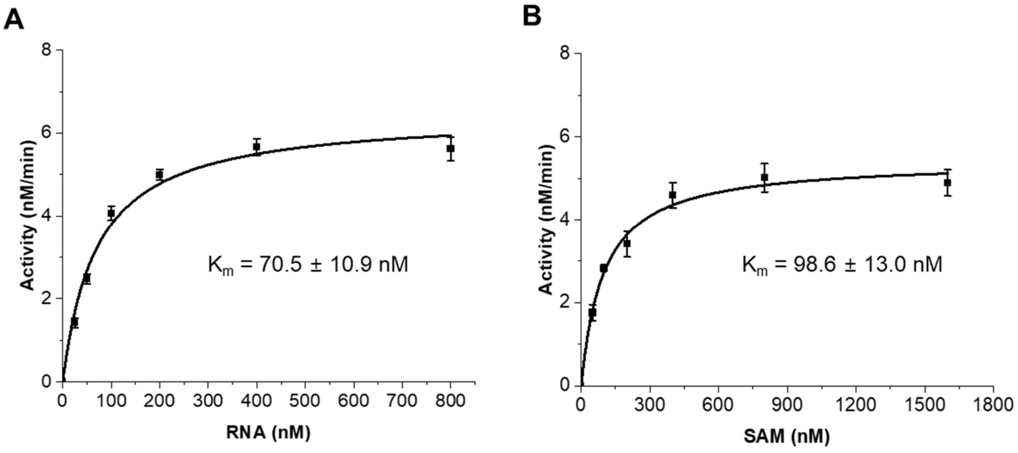

Figure S2. Confirmation of false-positive potential**.** (**A**) Confirmation of false positive potential that inhibit the coupled enzyme reaction process in which SAH is converted to ATP. (**B**) Confirmation of false positive potential by aggregation through centrifugation experiment.

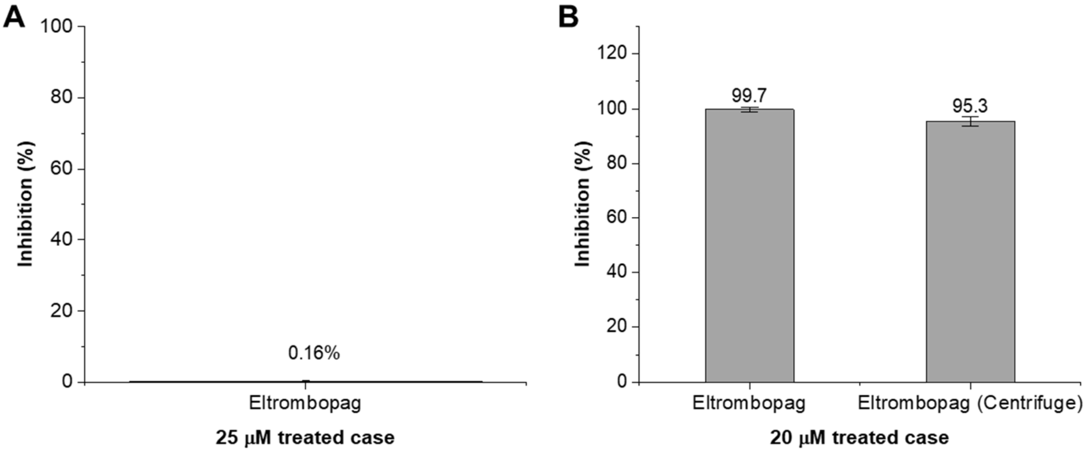

Figure S3. The anti-proliferative activity of gilteritinib as positive control was confirmed a GI_{50} value of 16.6 nM in MOLM-13.

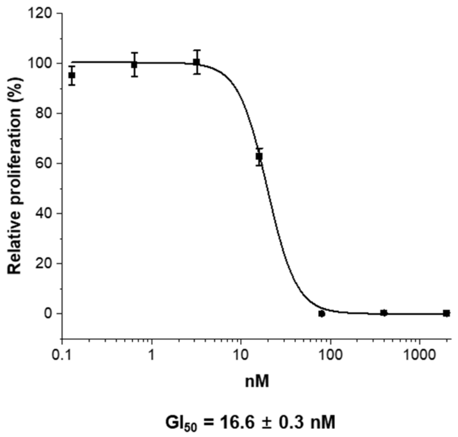

Figure S4. m^{6}A levels on poly-A+-enriched mRNA after 24h of eltrombopag treatment (40 μM) or 4 days after post-transduction of shRNA in MOLM-13

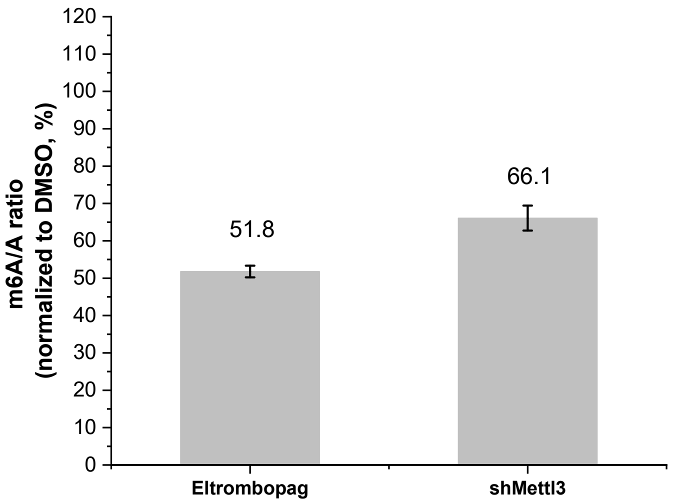

Figure S5. Analysis of combination treatment of AML drugs, gilteritinib and sorafenib, with eltrombopag in MOLM-13. (**A**) Dose-response matrix for the gilteritinib/eltrombopag combination in MOLM-13 cell line. (**B**) 2D synergy maps and calculated HSA synergy scores for the gilteritinib/eltrombopag combinations using Synergyfinder software. (**C**) Dose-response matrix for the sorafenib/eltrombopag combination in MOLM-13 cell line. (**D**) 2D synergy maps and calculated HSA synergy scores for the sorafenib/eltrombopag combinations using Synergyfinder software. Red and green areas represent synergy and antagonism, respectively. All experiments were repeated at least 3 times.

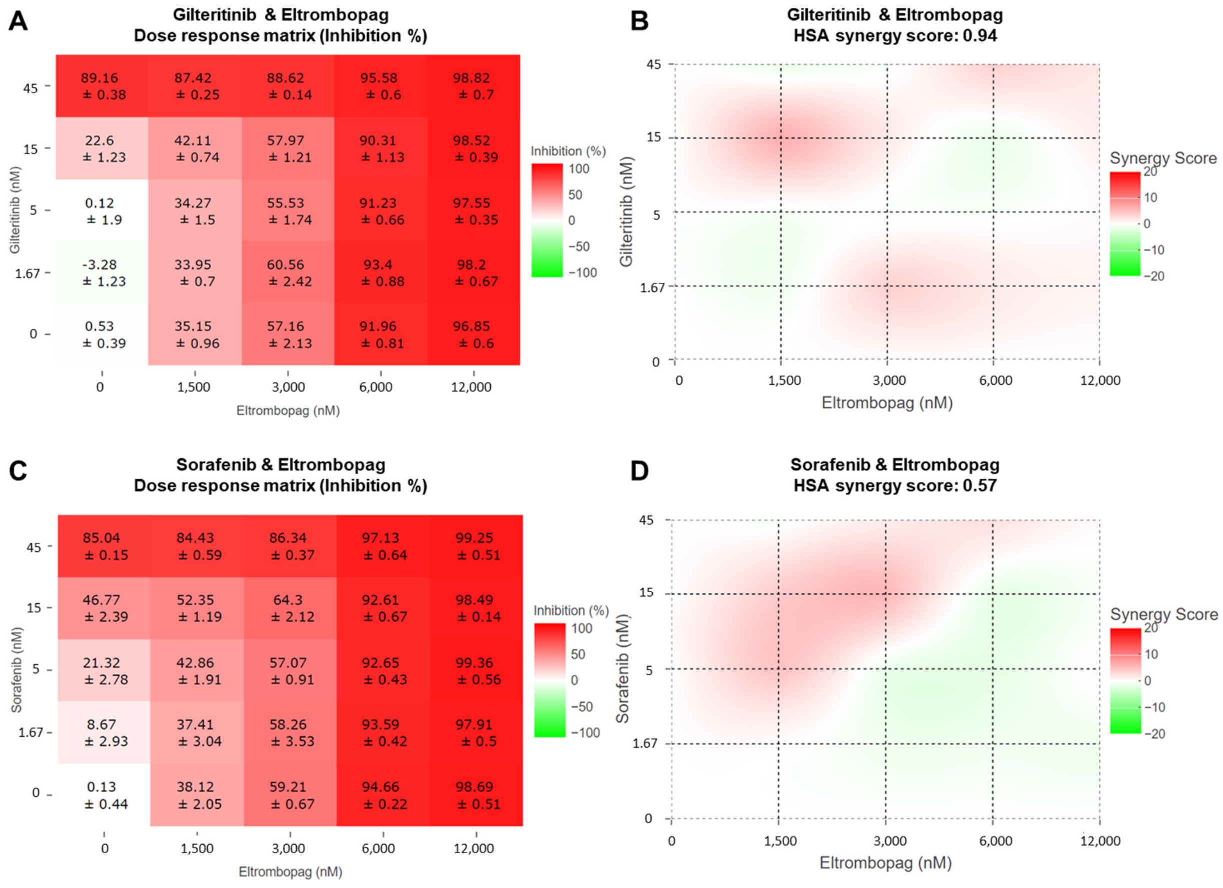

Figure S6. Distribution of significantly differential m^{6}A methylated peaks detected in shMETTL3-treated Molm-13 cells.

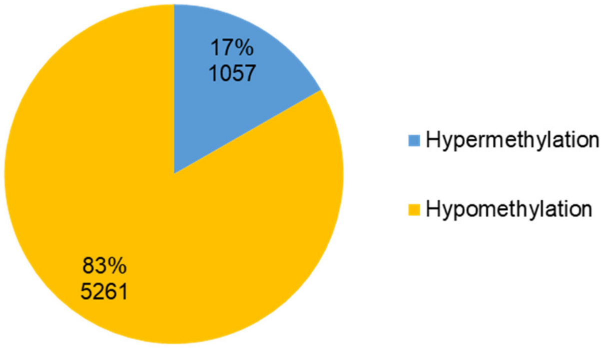

Figure S7. NMR spectrum of compound **14a**

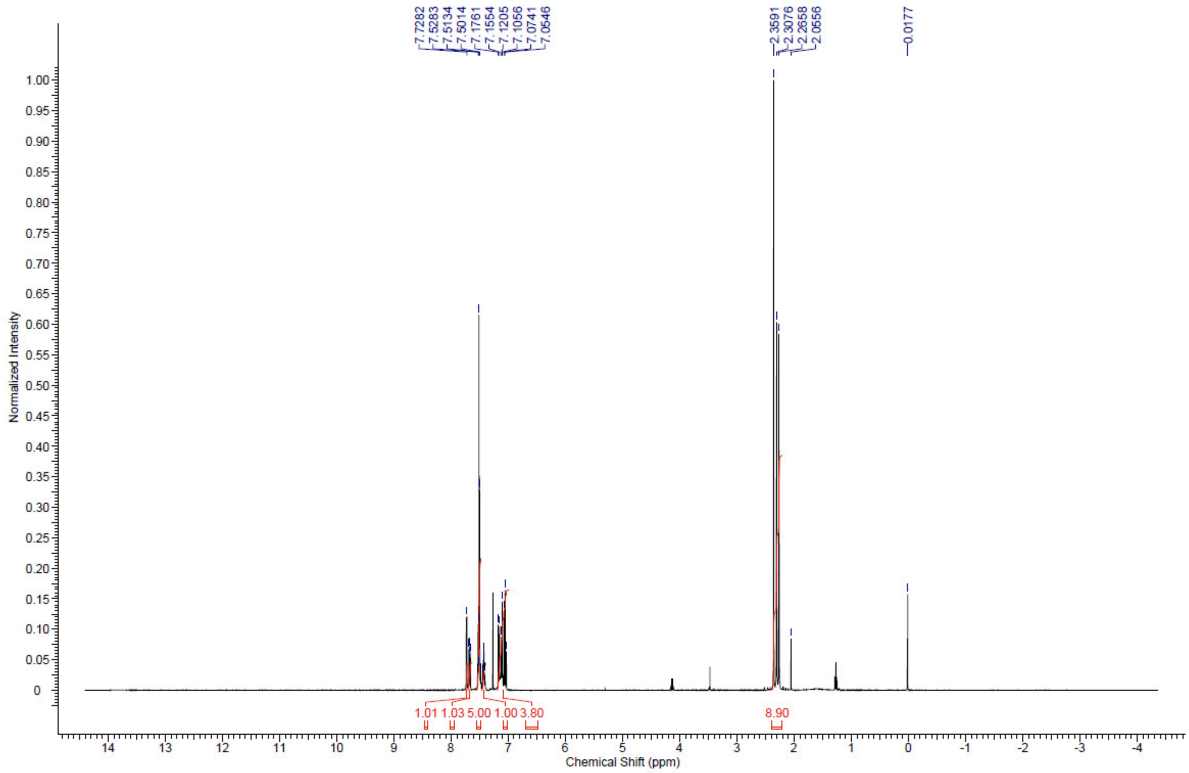

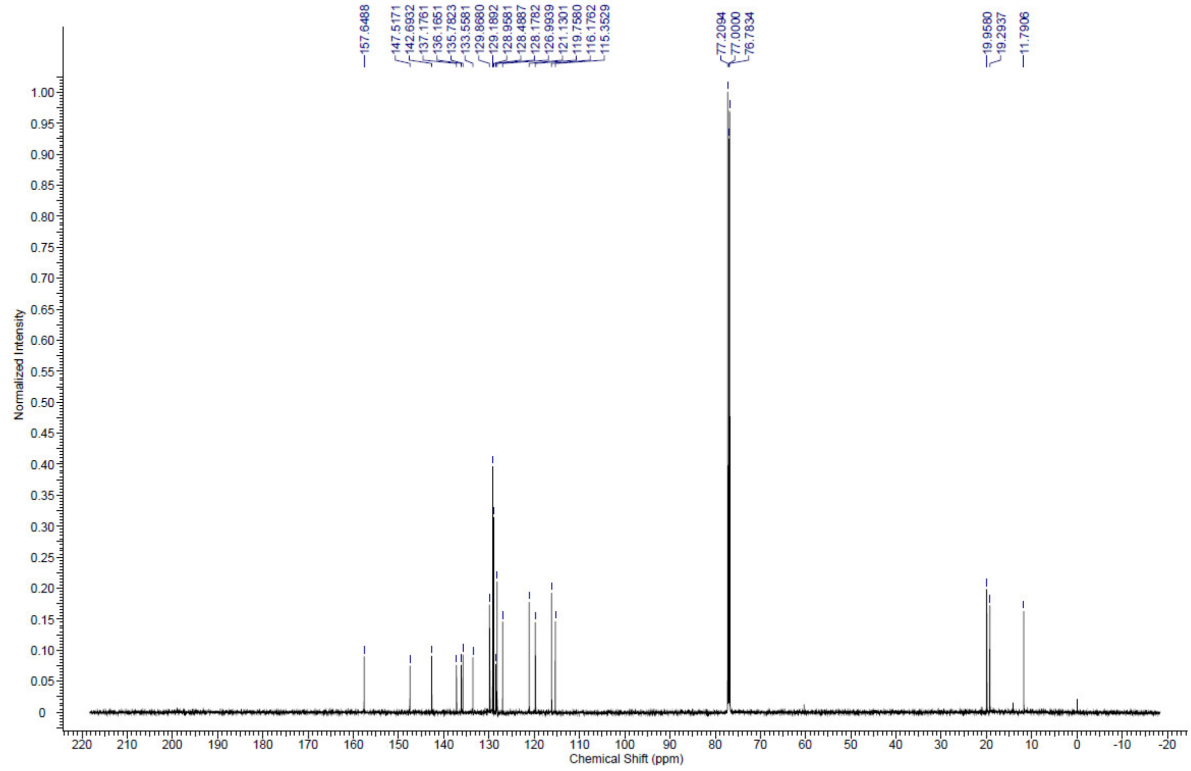

Figure S8. NMR spectrum of compound **14b**

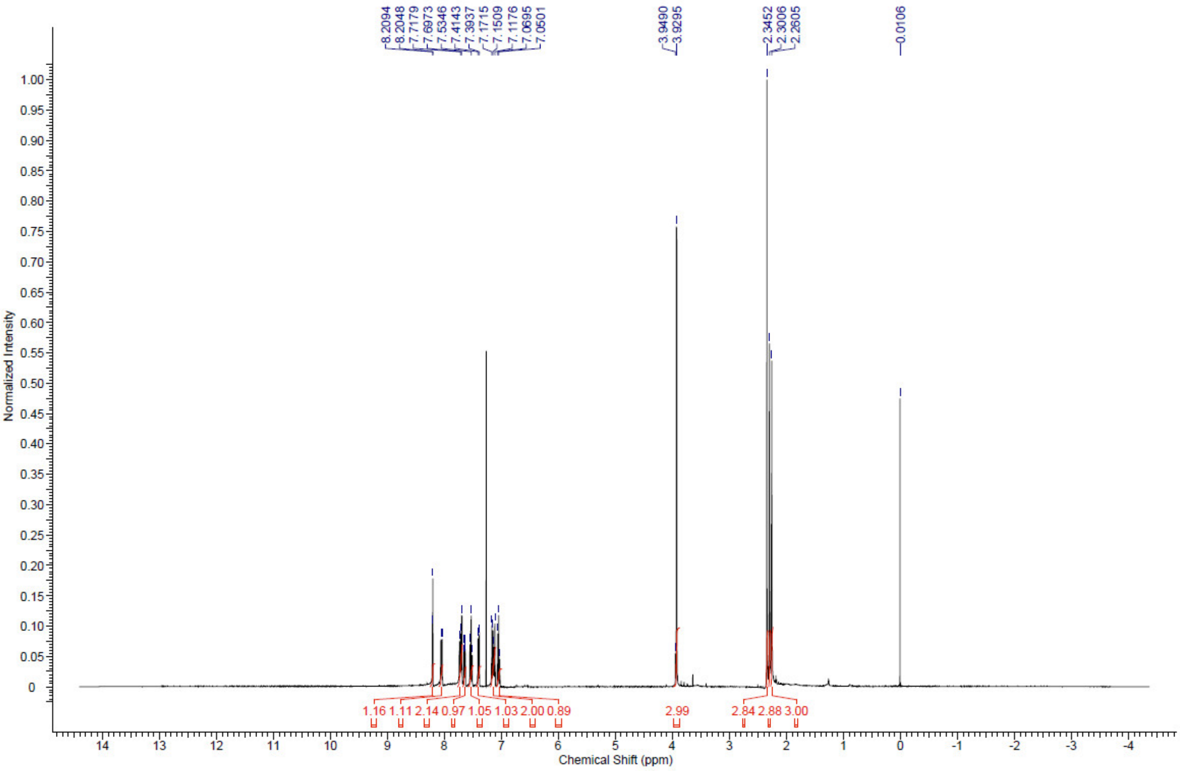

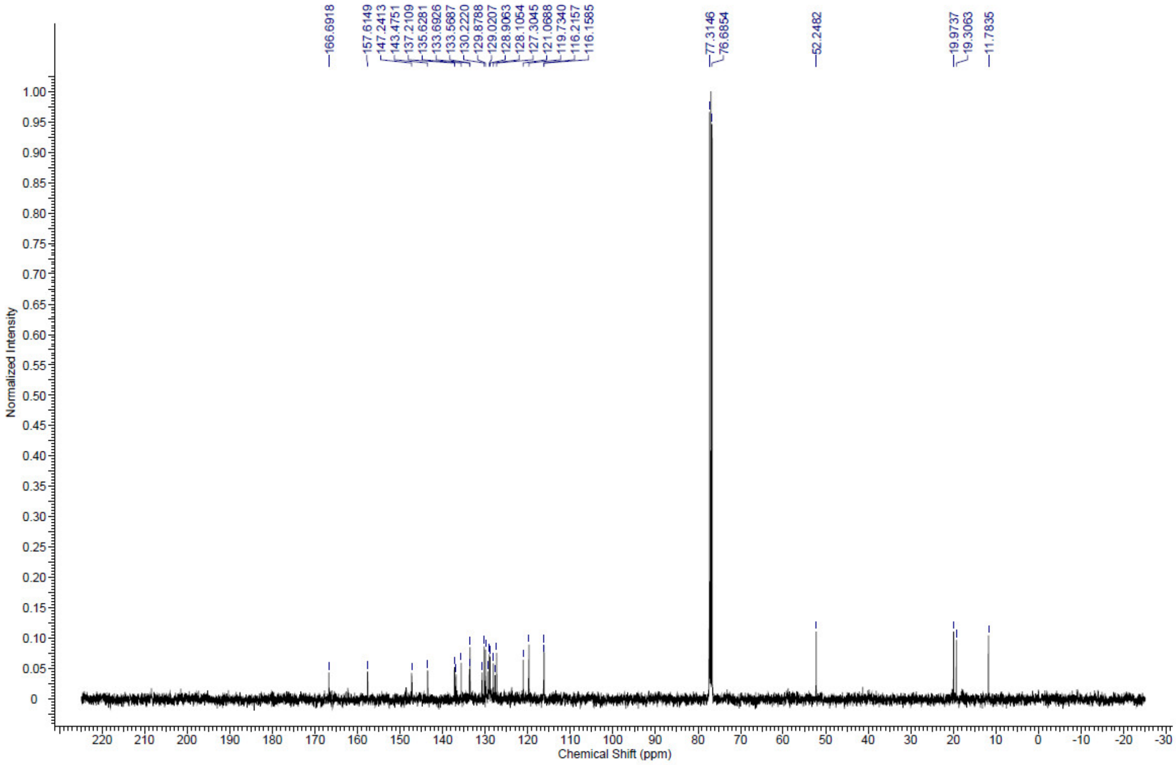

Figure S9. NMR spectrum of compound **14d**

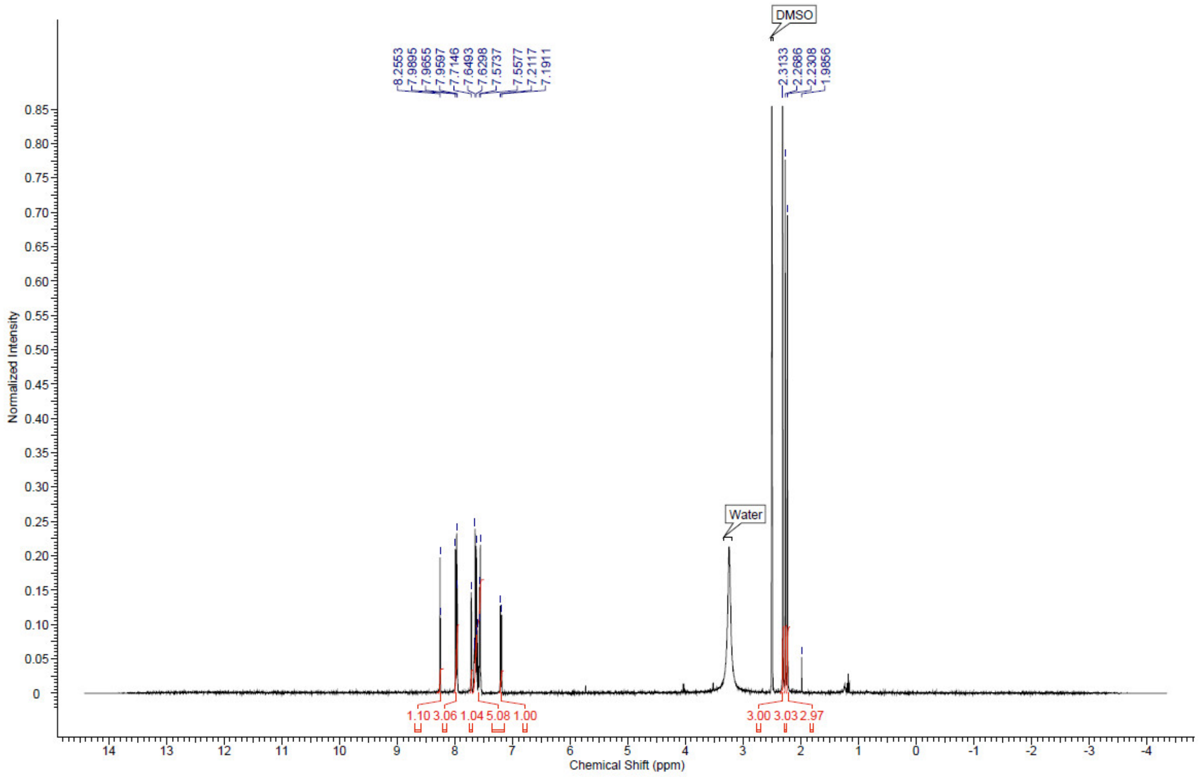

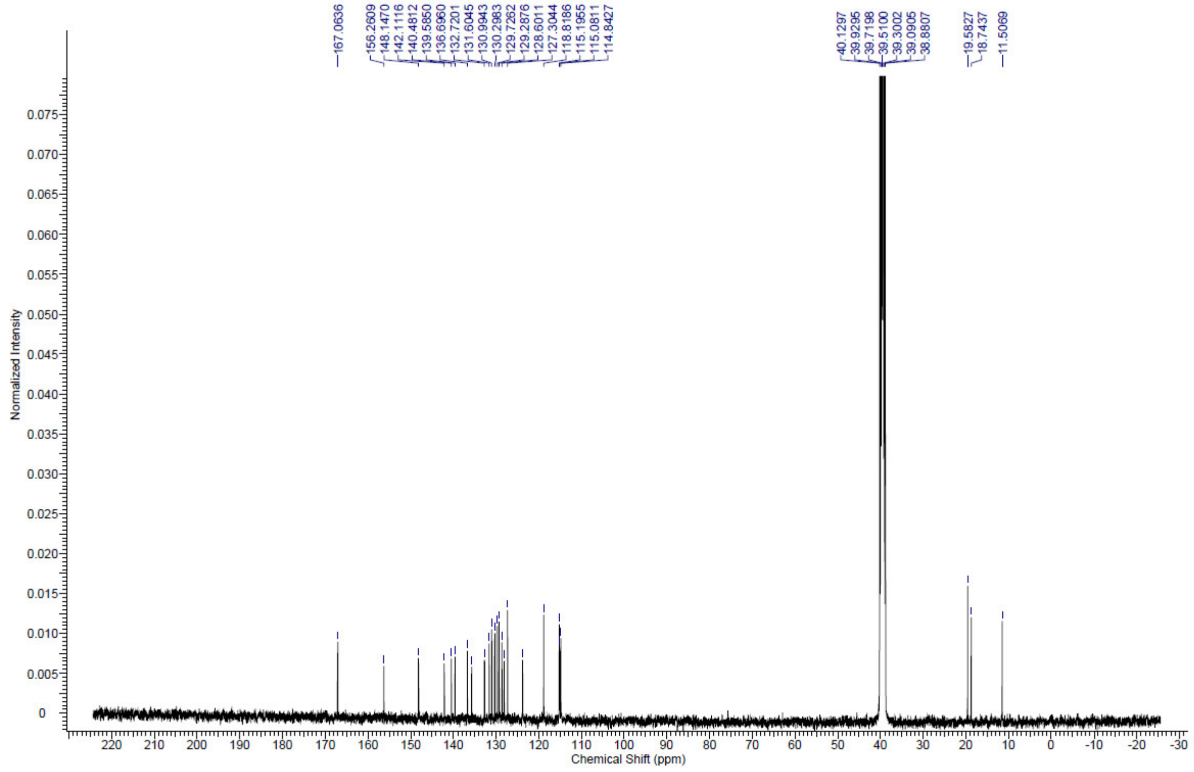

Supplement: Supplementary file 1 [file pharmaceuticals-15-00440-s001.zip › pharmaceuticals-1632371-supplementary/Revise_pharmaceuticals-1632371 - sup.pdf]
